# Pioneers in Dermatology and Venereology: An interview with Professor Michael Landthaler

**DOI:** 10.1111/jdv.20716

**Published:** 2025-06-25

**Authors:** Michael Landthaler

**Affiliations:** ^1^ Department of Dermatology University of Regensburg Regensburg Germany


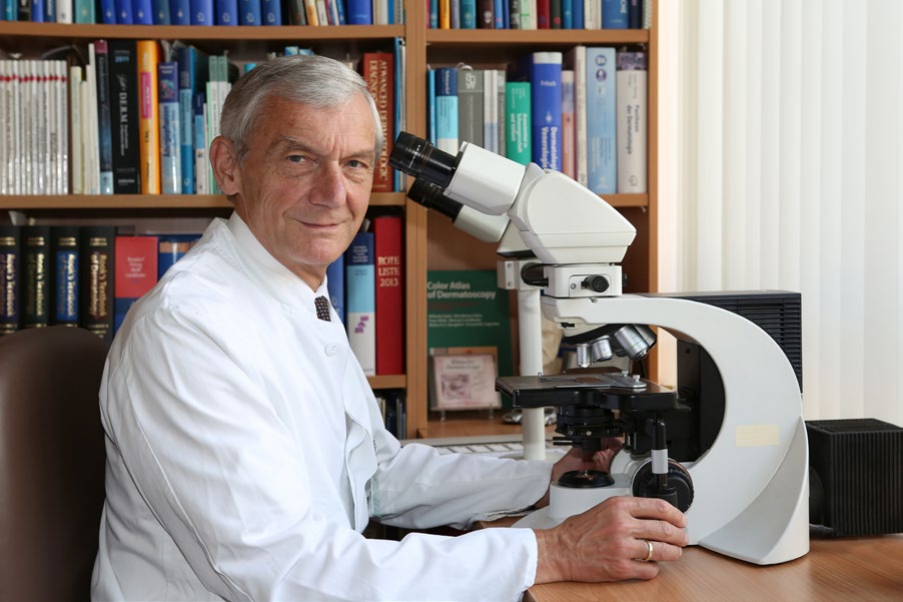


## CURRICULUM VITAE

Year of birth: 1948

## ACADEMIC EDUCATION

1967 to 1973 Medical studies at the Ludwig Maximilian University of Munich
1976 to 1991 Further training as a dermatologist at the Ludwig Maximilian University of Munich, Germany (Head of the Department: Professor Otto Braun‐Falco)
1984 Postdoctoral lecture qualification

## PROFESSIONAL CAREER

1988 Appointment to a C3 professorship at the Ludwig Maximilian University of Munich, Germany
1991 to 2013 Head of the Department of Dermatology at the University of Regensburg, Germany
1994 to 2000 Dean of the Medical Faculty of the University of Regensburg, Germany
2000 to 2002 Vice Rector of the University of Regensburg, Germany
2002 to 2012 Deputy Medical Director of the University Hospital Regensburg, Germany
2007 Honorary Doctorate by the Semmelweis University in Budapest
2013 Retirement

## SOCIETIES AND MEMBERSHIP

Since 2001 Member of the German National Academy of Sciences Leopoldina
1997 to 2013 Member of the Board of the German Dermatological Society and Treasurer in the Executive Committee from 2007 to 2013

## WHAT BROUGHT YOU TO DERMATOLOGY?

Professor Otto Braun‐Falco's dermatology lecture was among the most inspiring experiences I had in Munich. Coincidentally, my final state examination concluded with dermatology, and our group performed exceptionally well that the senior physician examiner offered each of us a position. I seized the opportunity to take up a position at the prestigious Munich clinic, and I quickly realized that dermatology was the medical specialty where I truly belonged.

## WHO WERE YOUR MOST IMPORTANT TEACHERS?

My most influential teacher was, of course, Professor Otto Braun‐Falco who led the Munich clinic. Other key mentors in my academic development included the senior physicians Professor Plewig, Professor H.H. Wolff, Professor Dorn, and Dr. Konz.

## FROM WHOM DID YOU LEARN MOST?

Naturally, I learned the most from Professor Otto Braun‐Falco, who embodied the academic triad of patient care, teaching, and research, setting a daily example for his team.

## WHAT ADVICE WAS MOST HELPFUL FOR YOUR CAREER?

When assuming a chair, one receives plenty of valuable advice on patient care, teamwork, teaching and more. I found meaningful guidance from the Rule of Saint Benedict of Nursia (480‐547 CE), whose principles resonate with the responsibility of managing a clinic and caring for patients. One example reads “*He should know that whoever undertakes the government of souls must prepare himself to account for them.*” Another passage says: “*It is not the healthy who need a physician, but the sick […] Care for the sick must rank before and above all else […] Let the sick, on their part, bear in mind that they are served out of honour for God, and let them not by their excessive demands distress anyone who serves them. Still, sick monks must be patiently borne with, because serving them leads to a greater reward.*” Finally, regarding the role of an abbot (comparable to the head of the clinic), I would like to quote: “*He should always remember what he is and what he is called, and should know that to whom more is committed, from him more is required*.”

## PLEASE LIST YOUR 5 BEST PUBLICATIONS

Landthaler, M et al Laser therapy of venous lakes (bean‐Walsh) and telangiectasias. J Plast Reconstr Surg 1984;73:78–81.
Maisch T, et al. The role of singlet oxygen and oxygen concentration in photodynamic inactivation of bacteria. Proc Natl Acad Sci USA. 2007;7223–7228.
Hafner C et al. Multiple oncogenic and clonal relationship in spatially distinct benign human epidermal tumours. Proc Natl Acad Sci USA. 2010;20780–20785.
Burgdorf W, Plewig G, Wolff HH, Landthaler M (eds). Braun‐Falco's Dermatolog. Springer (2009).
Schreml et al. 2D illuminescence imaging of pH in vivo. Proc Natl Acad Sci USA. 2011;2432–2437.
Groesser L, et al. Postzygotic HRAS and KRAS mutations cause nevus sebaceous and Schimmelpenning syndrome. Nat Genet. 2012;44:783–787.

## HAVE YOU EVER BEEN PRESIDENT OR IN THE LEADERSHIP OF AN ACADEMIC SOCIETY?

Yes, I served as Dean of the Medical Faculty at the University of Regensburg from 1994 to 2000, followed by a term as Vice Rector of the University from 2000 to 2002. I was also a Member of the Presidium of the German Dermatological Society from 2007 to 2013 and Deputy Medical Director of the University Hospital Regensburg from 2001 to 2011.

## WHAT WAS THE GREATEST ACHIEVEMENT IN YOUR PROFESSIONAL LIFE?

I had the opportunity to contribute to the development of dermatology in Regensburg, helping to establish a dermatology clinic, a university clinic and a medical faculty, which are now firmly integrated into the university structure. Furthermore, I am glad that three senior physicians from the clinic in Regensburg now lead dermatology clinics across Germany: Professor Stolz in Munich, Professor Vogt in Homburg/Saar and Professor Szeimies in Recklinghausen. I am particularly proud of the appreciation the junior doctors expressed upon my retirement with these words: “*In particular, we would like to thank you for instilling in us a medical ethos and approach that places the patient at the centre, with their individual wishes, fears and needs. We hope to preserve and carry forward these values in our future clinical work*.”

## WHAT WAS THE GREATEST DISAPPOINTMENT IN YOUR PROFESSIONAL LIFE?

The greatest disappointment was seeing well qualified young colleagues, with great potential for an academic career, leave the university to pursue work in a private practice.

## CAN YOU SHARE ANY FUNNY EPISODES FROM YOUR PROFESSIONAL LIFE?

In the 1980s, we were asked by a company to study the effects of an Nd:YAG laser on the skin. We chose mini‐pigs for the study, as their skin closely resembles that of humans. The experiments had to take place in a clinic, which was the only location equipped with the necessary infrastructure for laser treatment. The pig was anaesthetised at 7 am, and we proceeded as planned. However, the pig took longer than expected to wake up, and the vet refused to transport it while still under anaesthesia. To avoid alarming our patients, we covered the pig's cage as the patients’ treatment started at 9 am. Eventually, the pig woke up and we found ourselves treating patients while trying to manage a restless and grunting pig.

## WHAT DO YOU LIKE BEST IN YOUR PROFESSION?

The academic triad of patient care, research, and teaching was both a daily challenge and a source of satisfaction. Dermatology is also a clinical‐morphological discipline with a wide spectrum, encompassing areas such as conservative and surgical dermatology, allergology, oncology, proctology, andrology, and histopathology. Even 12 years after retiring, I continue to practice histopathology.

## WHOM WOULD YOU LIST AMONG THE TOP TEN DERMATOLOGISTS (PLEASE NAME ONLY DECEASED PERSONS)?

I would include Professor Otto Braun‐Falco, Professor Klaus Wolff, Professor Steve Katz, Professor Wolfram Sterry, Professor Leon Goldman, and Professor Bernard Ackerman.

## WHOM WOULD YOU LIST AMONG THE TOP TEN LIVING DERMATOLOGISTS?

Among them are Professor Georg Stingl, Professor Lars French and Professor Boris Bastian.

## APART FROM DERMATOLOGY, WHAT IS YOUR MAJOR INTEREST?

In addition to dermatology, I have a strong interest in European history, traveling and sports.

## WHO ARE YOUR FAVOURITE WRITERS, COMPOSERS, AND PAINTERS?

My favourite classical composers include Beethoven, Bach, Mozart, Hayden and Vivaldi, while my favourite contemporary composer is Ligeti. The emotional power of music can be felt in works such as Beethoven's *Missa solemnis*, Haydn's *Creation* and Vivaldi's *Four Seasons*.

I consider Michelangelo's *The Creation of Adam* and the *Pietà* to be among the most impressive masterpieces of classical art, along with Vincent van Gogh's *Portrait of Doctor Gachet*, whose expression captures the pain of his time. In his book *Fatum*, Kyle Harper explores the role of climate change and epidemics in the fall of the Western Roman Empire, drawing parallels with our modern world. Siddhartha Mukherjee's *The Emperor of Maladies: a biography of cancer* takes a biographical approach to cancer. It is a tale of suffering, the pursuit of research, creativity and perseverance, but also of the arrogance and venality of physicians.

## WHAT WAS YOUR LATEST DISCOVERY?

The most significant recent discovery for me has been the diversity of Europe, its history, its landscapes, its people and its many cultures, which I have been exploring more deeply in recent times.

## HOW WOULD YOU DEFINE A PERFECT DAY?

A perfect day is one where I can freely organise my time, something I have been fortunate enough to do for the past 12 years. Now, I have time for sports, reading, traveling, and spending time with my family, including my four grandchildren.

## WHAT WILL BE THE GREATEST PROBLEMS FOR DERMATOLOGY IN THE NEXT 10 YEARS?

The current challenges of the healthcare system, such as staff shortages, expensive modern medicines, over‐regulation, and extensive documentation requirements, will naturally also impact dermatology.

## WHAT WILL BE THE NEXT BREAKTHROUGHS IN THE COMING YEARS IN DERMATOLOGY?

New system therapies for inflammatory skin diseases, personalised medicine, advances in the treatment of malignant melanoma and, above all, the integration of artificial intelligence will significantly enhance the diagnosis and treatment of skin diseases.

## WHAT IS THE KEY MESSAGE YOU WANT TO GIVE YOUR YOUNGER COLLEAGUES?

I advise young colleagues to pursue the broadest possible clinical training, including surgical and systemic therapies, to commit to continuous professional development, and to follow to the principles of Saint Benedict: “*It is not the healthy but the sick who need a physician*.” At a time when the work‐life balance is so widely discussed, I would like to quote the Indian philosopher Rabindranath Tagore (1861–1941): “*I slept and dreamt that life was joy. I awoke and saw that life was service. I acted, and behold, service was joy*.”

## CONFLICT OF INTEREST STATEMENT

None declared.

*Note: *The Pioneers in Dermatology and Venereology* interview was conceived and conducted by Johannes Ring.

